# Design and construction of a photobioreactor for hydrogen production, including status in the field

**DOI:** 10.1007/s10811-016-0789-4

**Published:** 2016-01-27

**Authors:** Kari Skjånes, Uno Andersen, Thorsten Heidorn, Stig A. Borgvang

**Affiliations:** Norwegian Institute of Bioeconomy Research—NIBIO, PO 115, N-1431 Ås, Norway

**Keywords:** Photobioreactor, Microalgae, Hydrogen, Bioenergy

## Abstract

Several species of microalgae and phototrophic bacteria are able to produce hydrogen under certain conditions. A range of different photobioreactor systems have been used by different research groups for lab-scale hydrogen production experiments, and some few attempts have been made to upscale the hydrogen production process. Even though a photobioreactor system for hydrogen production does require special construction properties (e.g., hydrogen tight, mixing by other means than bubbling with air), only very few attempts have been made to design photobioreactors specifically for the purpose of hydrogen production. We have constructed a flat panel photobioreactor system that can be used in two modes: either for the cultivation of phototrophic microorganisms (upright and bubbling) or for the production of hydrogen or other anaerobic products (mixing by “rocking motion”). Special emphasis has been taken to avoid any hydrogen leakages, both by means of constructional and material choices. The flat plate photobioreactor system is controlled by a custom-built control system that can log and control temperature, pH, and optical density and additionally log the amount of produced gas and dissolved oxygen concentration. This paper summarizes the status in the field of photobioreactors for hydrogen production and describes in detail the design and construction of a purpose-built flat panel photobioreactor system, optimized for hydrogen production in terms of structural functionality, durability, performance, and selection of materials. The motivations for the choices made during the design process and advantages/disadvantages of previous designs are discussed.

## Introduction

In the early 1940s, Hans Gaffron and his group observed that *Chlamydomonas reinhardtii* had the ability to produce hydrogen under anaerobic conditions (Gaffron and Rubin 1942). The production of hydrogen from water and sunlight using microalgae has been the subject of applied research since the early 1970s (Benemann 1996), but since the discovery of hydrogen production by sulfur deprivation (Ghirardi et al. 2000; Melis et al. 2000), which allowed a significant amount of hydrogen to be produced from light by biophotolysis, this research has advanced substantially (Burgess et al. 2011; Eroglu and Melis 2011; Dubini and Ghirardi 2015; Gonzalez-Ballester et al. 2015).

The fact that any exploitation of microalgae should be assessed and evaluated as a complete process rather than focusing solely on isolated elements of the process is a point that gains increasing recognition (Wijffels et al. 2010; Skjanes et al. 2013; Fresewinkel et al. 2014). However, topics such as the design of photobioreactors and associated challenges are examples of highly relevant ongoing developments.

Research related to the choice of photobioreactors, performance, and design has been published by numerous research groups over the last two decades. The advantages and disadvantages of the various photobioreactors systems developed have been assessed, and improvements suggested and tested (Barbosa et al. 2004, 2005; Kunjapur and Eldridge 2010; Harris et al. 2013; Pereira et al. 2014; Li et al. 2015; Heining and Buchholz 2015). Today’s increasing involvement of commercial interests in microalgae research and biomass production entails that focus is often on pilot- to large-scale facilities, as well as on decreased production costs.

Although research on microalgae has increased over the last years, with the number of publications on microalgae doubled and the number of publications on photobioreactors tripled (Bosma et al. 2014), there is still a need for further laboratory research related to the choice and performance of photobioreactors for various purposes in lab scale. One specific purpose which involves certain challenges is the abovementioned hydrogen production.

This paper summarizes the existing experiences within the use of different photobioreactor designs for hydrogen production from microalgae, both lab-scale and upscaling attempts. A selected design for both cultivation and production of hydrogen in lab scale is described in detail.

## Photobioreactors for hydrogen production

A high number of photobioreactor designs have been used for microalgae cultivation (Borowitzka 1999; Molina Grima et al. 1999; Posten 2009; Morweiser et al. 2010; Chen et al. 2011; Olivieri et al. 2014), and many of these have also been explored for use in hydrogen production studies (Dasgupta et al. 2010). These designs can be grouped into seven main design principles. Below is a summary of these main design groups, with examples of how the different photobioreactors have been used for hydrogen production from green microalgae. Agitation methods have been given particular attention since agitation of the culture is an important feature in photobioreactors for hydrogen production. The method for hydrogen production from green microalgae using sulfur deprivation has recently been reviewed, including upscaling attempts (Torzillo et al. 2015). Light conversion efficiency and hydrogen production efficiencies for phototrophic organisms, and the relations to bioreactor design, have previously been reviewed (Akkerman et al. 2002).

### Shaking flasks

The most simple setup in use for microalgae cultivation, having also been used for hydrogen production, is Erlenmeyer flasks with shaking as the agitation method. This has for example been applied for studies of cell age optimization for hydrogen production (Kim et al. 2005), for studies of the effect of light intensity on hydrogen production from *Chlamydomonas* (Sim et al. 2005), and for studies of hydrogen production from *Tetraselmis* (*Platymomonas*) (Guan et al. 2004). A disadvantage of using a culture vessel with movement as the agitation method is that it can make continuous gas collection more challenging.

### Stirred tank (STR)

Different variations of stirred tank reactors represent a common category of photobioreactor used for hydrogen production at lab scale. The simplest systems consist of bottles with magnet stir bars, without inserted sensors, where extraction of samples from the bottles is needed to follow other parameters than hydrogen production through the experiment. The advantage of such simple systems is that they are very cheap and easy to handle, allowing a large number of parallel cultures to be studied. Equivalent bottles with sensors usually inserted from the top or from the side, allowing logging of several parameters inside the reactor, are also in common use.

The agitation method in the stir tank type of bioreactor is the use of magnetic stir bar or impeller. The temperature can be controlled by methods such as a circulating water jacket, water bath, heating or cooling coils, or fans. The advantages of such systems are that they are simple to handle in small scale, there can be many possibilities for insertion of a high number of sensors, efficient mixing makes the culture very homogenous, and in some formats internal illumination is possible. The disadvantages are low surface to volume ratio and high energy consuming agitation, and the fact that they are inconvenient both for outdoor cultivation and upscaling.

The process of producing hydrogen from the green alga *C. reinhardtii* by sulfur deprivation was first discovered by Melis and his group (Melis et al. 2000), who used 1-L flat Roux-type bottles with magnetic stirring. This setup has later been adopted by several groups around the world. It is still in common use, either with off-the-shelf bottles with a silicon rubber plug on the top or custom-made varieties with inserted sensors (Kosourov et al. 2002). Flat Roux-type bottles were for instance used to explore strain diversity, using stir bars at the bottom (Meuser et al. 2009) and on the side (Skjånes et al. 2008). These types of bottles were recently used in experiments simulating outdoor conditions (Oncel et al. 2015). A Roux-type bottle was also used to study the effects of the fluid dynamics on hydrogen production (Giannelli et al. 2009), where stirring was provided by a multiple impeller design driven by a magnetic stirrer.

Cylindrical bottles with magnetic stirring were used for dilution methods for prolonged hydrogen production (Laurinavichene et al. 2002), for studying starch metabolism and e-pathways during hydrogen production (Chochois et al. 2009), and for studying culture parameters during hydrogen production from *Chlorella* (Alalayah et al. 2015). Similar setups using Erlenmeyer flasks with magnetic stirring also have been used (Hahn et al. 2004; Timmins et al. 2009; Volgusheva et al. 2013). Cylindrical bottles were used for hydrogen production after a precultivation step in a vertical tube sparging photobioreactor outdoors (Geier et al. 2012). Simple bottles with impeller stirring are also sometimes used for hydrogen production (Antal et al. 2003).

The first report on hydrogen production from *C. reinhardtii* cultivated under autotrophic conditions used a cylindrical vessel with impeller stirring run by a magnetic stirrer (Tsygankov et al. 2006). A 1.5-L torus-shaped reactor with impeller stirring has been used for studying hydrogen production from both *Tetraselmis* (*Platymomonas*) and *C. reinhardtii* (Degrenne et al. 2010; Fouchard et al. 2008; Ji et al. 2010). Semicontinuous hydrogen production has been conducted for 167 days in 1-L flasks with impeller run by a magnetic stirrer (Fedorov et al. 2005) and for 127 days in 2.5-L tanks with impeller stirring (Oncel and Vardar-Sukan 2009). A vertical column with a magnetic stirrer was used for hydrogen production using cultures pregrown outdoors (Martin del Campo et al. 2014).

### Horizontal tubular

The horizontal tubular type photobioreactor is a design that is common for upscaled cultivation of microalgae, due to high productivity (Slegers et al. 2013). The horizontal tubes are usually quite thin, giving a high surface to volume ratio, and can be stacked in fence-like structures to give an optimal light capture efficiency for minimal use of land area. Temperature control in these structures can sometimes represent a challenge. In Nordic climate, this is often solved by placing the bioreactors in temperature-regulated greenhouses. When used in warmer climate, this type of bioreactor can be cooled by water spraying. Horizontal tubular reactors may also be placed on the ground, making water submersion an efficient method for temperature regulation. Agitation of the culture in horizontal tubular reactors is achieved by circulating the culture through the tubes using pumps. The disadvantages of using this type of reactors are the low gas exchange and a significant gas gradient along the tubes caused by most of the gas exchange being conducted in a separate chamber. Other disadvantages are high energy input and sometimes accumulation of biomass in the tubes (Molina et al. 2001; Xu et al. 2009; Posten 2009; Slegers et al. 2013).

Hydrogen production in 50-L horizontal tube reactors has been studied in outdoor environment and compared to indoor experiments (Scoma et al. 2012). A significant difference in hydrogen production efficiency was observed; however, a number of experimental differences could explain the variance. A compact 110-L reactor with horizontal tubes was submerged in liquid with light scattering nanoparticles and used for hydrogen production (Giannelli and Torzillo 2012). Another study for pilot-scale outdoor production of hydrogen used modeling for prediction of parameter influence (Vargas et al. 2014).

### Tubular coiled

A tubular coiled photobioreactor has many similarities to the horizontal tube reactor type (Lindblad et al. 2002). The agitation is obtained by pumps circulating the culture and bubbling by air/CO_2_, and the temperature can be controlled by water spraying or using heating or cooling coils. The advantages are high surface to volume ratio and the fact that the light harvesting efficiency per land area can be increased by a conic helical shape. The disadvantages of this type of reactor are the low gas exchange, high shear stress, accumulation of biomass in the tubes, and high energy input. The tubular coiled reactor type has not been extensively studied for use in hydrogen production, but a comparison of hydrogen production from this reactor type vs. a flat panel reactor has been performed (Oncel et al. 2014). The study suggested that although the tubular reactor was more efficient for cultivation, the flat panel design was a better choice for hydrogen production due to factors such as a more efficient removal of the produced gas.

### Vertical tubular (bubble column/airlift)

Photobioreactors constructed of vertical tubes are usually agitated by sparging of air; for exceptions, see “Stirred tank” section. For temperature control in outdoor systems, water spraying is the most common for this bioreactor type, but heating-cooling coils or water jacket has also been used and the latter is more common in lab-scale systems. The advantages of such a system are the efficient gas exchange, low shear stress, good mixing, low energy consumption, and low cost. The disadvantages are the low surface/volume ratio and inefficient solar radiation capture during outdoor cultivation. These designs are inconvenient for hydrogen production, since agitation of the culture depends on either gas addition leading to dilution of the produced hydrogen or gas circulation leading to higher risk of leakages.

### Immobilization

Microalgae can be immobilized in matrixes such as alginate beads, biofilm on solid surfaces, or in alginate films. This novel format has many advantages compared to the conventional liquid cultures. The films can consist of a very thin layer of cells, giving an outstanding surface to volume ratio and high cell density combined with a very short light path. There is no shear stress, the cells are protected inside the gel, and the cells can also be harvested in a simple manner. For hydrogen production, the fact that the nutrient availability can be easily altered represents an important aspect of this method.

Hydrogen production from immobilized microalgae was first demonstrated in a system where the algae were immobilized on a glass fiber matrix (Laurinavichene et al. 2006). Immobilization in alginate films provided a protective matrix where the algae produced hydrogen for a prolonged period, and a continuous production for 90 days was achieved by supplying limiting amounts of sulfur (Laurinavichene et al. 2008). Microalgae encapsulated in alginate films have shown a higher tolerance against O_2_ inactivation of hydrogen production, possibly due to slower diffusion of O_2_ through the alginate matrix (Kosourov and Seibert 2009). Algae immobilized in alginate films were also used to show increased production of hydrogen during different light/dark periods (Rashid et al. 2011) and using different C-sources and varying pH (Rashid et al. 2013). Hydrogen pressure inhibition of hydrogen production efficiency has been shown in immobilized cultures (Kosourov et al. 2012). Successful hydrogen production from immobilized cyanobacteria and other phototrophic bacteria has also been shown (Leino et al. 2012; Liao et al. 2015).

### Flat panel

Flat panel photobioreactors have many advantages and are commonly used for lab-scale studies of algae cultivation; upscaled versions have also been used outdoors. The advantages of this type of photobioreactors are a high surface to volume ratio and the fact that the light path can be very short and evenly distributed across the reactor. This bioreactor type can also have flexibility as regards light capture angle, and depending on which agitation method is used, a high mixing rate can be achieved in addition to low shear stress. Agitation methods used include sparging, which is the most common choice when used for algae cultivation. Other methods are magnetic stirring, impeller stirring, baffles, and rocking motion. Methods for temperature control include inserted or external heating/cooling coil, water circulation in a separate surface compartment, and heat exchanger on the surface of the reactor. The disadvantages in respect to hydrogen production are related to agitation methods. Agitation by sparging involves either dilution of the hydrogen gas or recirculation of the produced gas, which may lead to higher risk of leakages. Agitation by stirring using either magnetic stirring or impeller requires a high energy input. Agitation by rocking motion also requires energy, but possible approaches for practical use include using wave motion by positioning the reactor on the sea surface, possibly also including additional wind energy (Otsuki et al. 1998).

A study using horizontal flat panel photobioreactors with magnetic stirring found inhibiting effects from hydrogen headspace concentration on hydrogen production efficiency (Kosourov et al. 2012). Flat plate bioreactors have been used for hydrogen production with agitation by sparging with recirculated gas, avoiding hydrogen inhibition by using a sophisticated system for separating the hydrogen from the rest of the gas phase by Tamburic et al. (2011). As mentioned above, a comparison between a tubular coiled reactor and a flat panel reactor with impeller agitation was made (Oncel and Kose 2014), and it was concluded that the flat panel reactor was better suited for hydrogen production, mainly due to the fact that it could avoid the backpressure of accumulated hydrogen. A theoretical simulation to study the effect of the light path on hydrogen production has been made (Berberoglu and Pilon 2010). The flat plate horizontal rocking motion format, first described for algae cultivation (Davis et al. 1953), has been explored for hydrogen production from phototrophic bacteria (Gilbert et al. 2011).

In the following, the design and construction of a dual purpose, flat panel rocking motion photobioreactor is described. The reactor is designed for the purpose of algae cultivation and hydrogen production; experimental data from using the reactor will be published later.

## A selected design

### Criteria for selection

There are a large number of possible designs of photobioreactors suitable for microalgae cultivation. However, the number of previously explored designs for photobioreactors used for hydrogen production is much more limited, as described above.

The main purpose of the work presented herein was to construct a photobioreactor to be used for both microalgae cultivation and hydrogen production, fulfilling a number of different criteria as provided below.

The reactor was to be used for lab-scale experiments, with a bench top format easily operated in a lab environment. The ideal culture compartment size should be large enough to be suitable for many experimental setups, meaning it should be possible to use a sufficient culture volume to allow for regular culture sample extractions for measuring a number of important parameters during the experiments.

The reactor should optimally have qualities like even light path and efficient gas exchange for microalgae cultivation. A flat panel bubbling bioreactor is an attractive, well-explored option for microalgae cultivation due to these specific features. However, the addition of gas is an inconvenient agitation method when the reactor is used for hydrogen production since a dilution of the produced gas represents a large disadvantage. Circulation of the gas is also inconvenient due to possible increased risk of leakage. The possibility of using a flat panel design in a horizontal position where a rocking motion is used for agitation has been described previously (Davis et al. 1953; Soeder et al. 1981), and this option can be converted to a hydrogen production unit (Gilbert et al. 2011). The gas exchange is also important during the hydrogen production phase, which requires a sufficient velocity and angle of the rocking motion for agitation.

The bioreactor should also have the possibility for insertion of multiple sensors and electrodes for logging and control of multiple culture parameters. The bioreactor design should have a simple, low cost solution for temperature control and has low bench space requirements. The materials should be solid enough to tolerate intensive use, taking into account characteristics such as strength of threads of ports and openings, avoiding tension in breakable materials such as glass caused by contact between these materials and harder materials such as metals.

A very important criterion for an optimal design and use of a photobioreactor for hydrogen production is to prevent all hydrogen leakage to the extent possible. This means that all materials in contact with hydrogen gas or a hydrogen producing culture should have properties assuring minimal penetration of hydrogen gas. In addition, all connections should have seals proved to be hydrogen leakage free, both by material properties and by positioning of the seal. The design should allow for simple repeated sample extractions from both culture, collected gas, and headspace of the culture compartment, without loss of hydrogen tightness properties.

For lab-scale monoculture experiments, the possibility to autoclave the system represents an important feature. For some designs, this may represent a challenge. The parts of the bioreactor exposed to the culture have to be autoclavable without loss of hydrogen tightness properties.

The photobioreactor system should also include a control system for controlling multiple parameters such as temperature, pH, optical density (OD), CO_2_ supply and agitation, including also logging of parameters such as dissolved oxygen (DO) and produced gas.

The flat panel rocking motion bioreactor type was selected due to the potential of fulfilling all the criteria described above. The design allowed for use in two modes: the first where the flat panel culture vessel was placed in a vertical position, and a gas sparging system for the addition of air/CO_2_ was used for agitation and gas exchange. In the second mode, the flat panel vessel was tipped over into a horizontal position and a motor-driven rocking motion was used for agitation. The first mode was used for algae cultivation and the second mode for hydrogen production. A custom-made gas collection system was designed for the collection of the produced hydrogen gas, addressing the challenges of collecting hydrogen from a vessel in movement. A custom-made control system was also designed.

The actual design of the photobioreactor is described in detail below.

### Materials

All materials used for the different components of the photobioreactor were assessed against criteria such as the following:

- Heat resistance, autoclavability
- Resistance against oxidation/corrosion
- Durability
- Availability on the market
- Hydrogen leakage
- Weight
- Transparency whenever needed
- Potential adverse effects on the microalgae cultures

These materials are listed and assessed below, i.e., metals for inner and outer bioreactor frames, coating, glass types, silicon, gaskets, and O-rings.

#### Metals

The inner frame was constructed of stainless steel, a material with high strength, easily molded. The alloy SS AISI 316L (Table 1, material 1) was chosen based on its resistance against corrosion. Many parameters affect the corrosion tendency of steel alloys, such as temperature, pH, and salinity. In cases where the photobioreactor is used to cultivate marine strains, the salinity combined with heat during autoclaving will influence the corrosion resistance significantly. To our knowledge, there are currently no alloys of stainless steel available completely resistant to corrosion. Protection of the stainless steel against corrosion is one of the advantages with the Teflon coating described below.

**Table 1:** Materials used in the photobioreactor, with advantages and disadvantages

Part	No.	Material	Advantage	Disadvantage
Inner frame	1	Stainless steel AISI 316L	– High strength – Less corrosive than other SS alloys	– Heavy weight – Alloy contains the potential toxic elements Cr, Ni, and Mo – Potentially corrosive
Inner frame coating/coating of bolts	2	Primer: Primer Black 420–710 (Du Pont) Outer coating: PFA Powder Sparkling Clear 532–7000 (Du Pont)	– Inert – Ease of cleaning – Replaces lubricant for O-rings – Protects steel from culture exposure, reducing the risk of corrosion – Resistant to autoclaving – Protects algae against metal exposure – Prevents algae attachment	– Sensitive to scratches
Outer frame	3	Aluminium alloy 5052 H32	– Light weight – Sufficient strength – Less corrosive than other aluminium alloys	
Glass windows	4	Fortified soda-lime glass	– High transparency – High strength	– Strength sensitive to jagged edges
Bolts	5	Stainless steel 316	– High strength	
Gasket between glass sides and outer frame	6	Silicon rubber 40SH	– Protects against tension between glass and metal	
O-ring seal between inner frame and glass sides	7	Viton FKM 70 shore (Eriks)	– Hydrogen tight	– Needs frequent replacement
O-ring seal between inner frame and bolts, electrodes and ports	8	Viton FKM 75 shore (Eriks)	– Hydrogen tight	– Needs frequent replacement
Septa of sampling ports	9	GR-2 silicon rubber (Supelco)	– Hydrogen tight – Self-sealing after needle penetration – Low cost	– Needs frequent replacement
Gas collection unit	10	Borosilicate glass	– Transparent	– Breakable
Exhaust tube for gas during bubbling phase	11	Borosilicate glass	– Transparent	– Breakable
Inlet tube for air/CO_2_ mixture Outlet tube for water displacement from gas collection unit	12	Silicone peroxide, cross-linked (VWR)	– Transparent – Very flexible	– Cannot be exposed to hydrogen, due to high hydrogen penetration

The outer frames were constructed of aluminium alloy 5052 H32 (Table 1, material 3), a lightweight material with sufficient strength for its purpose. This alloy is more resistant toward corrosion than other aluminium alloys. It was particularly important to use a lightweight metal for the outer frame, as the steel alloy of the inner frame made the reactor quite heavy.

#### Coating

There are many materials commonly used for building photobioreactors that could potentially have inhibiting effects on the algae (see the inhibition study described below). A protective coating could have shielding effects in this respect, in addition to its many other advantages (see Table 1). The inner frame, made from stainless steel containing the potential harmful elements Cr, Ni, and Mo, was coated with the fluoropolymer perfluoroalkoxy polymer resin (PFA) (Table 1, material 2), a material belonging to the group of materials commonly called Teflon. Many PFA resins contain color. This is due to the increased convenience of application. However, even if the composition of PFA resins often is protected by IP rights and therefore has no composition declarations, there are indications that these color components frequently contain certain metal oxides which, if released from the material, could potentially have harmful effects on the algae. A colorless composition was chosen for the photobioreactors described herein, with a black primer acting as a base attaching the PFA resin to the metal. In addition to protecting the microalgae from the metal, the PFA coating prevents corrosion of the stainless steel by protecting the metal from the algae culture. The coating also has properties that can be used to replace any lubricant, often necessary to make a gas-tight seal between O-rings and metal. Furthermore, the PFA makes the frame easier to clean.

#### Gaskets

A flat gasket made from silicon rubber (Table 1, material 6), 1 mm thick, was cut to fit the outer edge of the glass and form a 10-mm-wide padding between the glass and the outer frame. The function of this gasket was to prevent tension in the glass caused by contact between the glass and the aluminium frame.

**Fig. 1:**
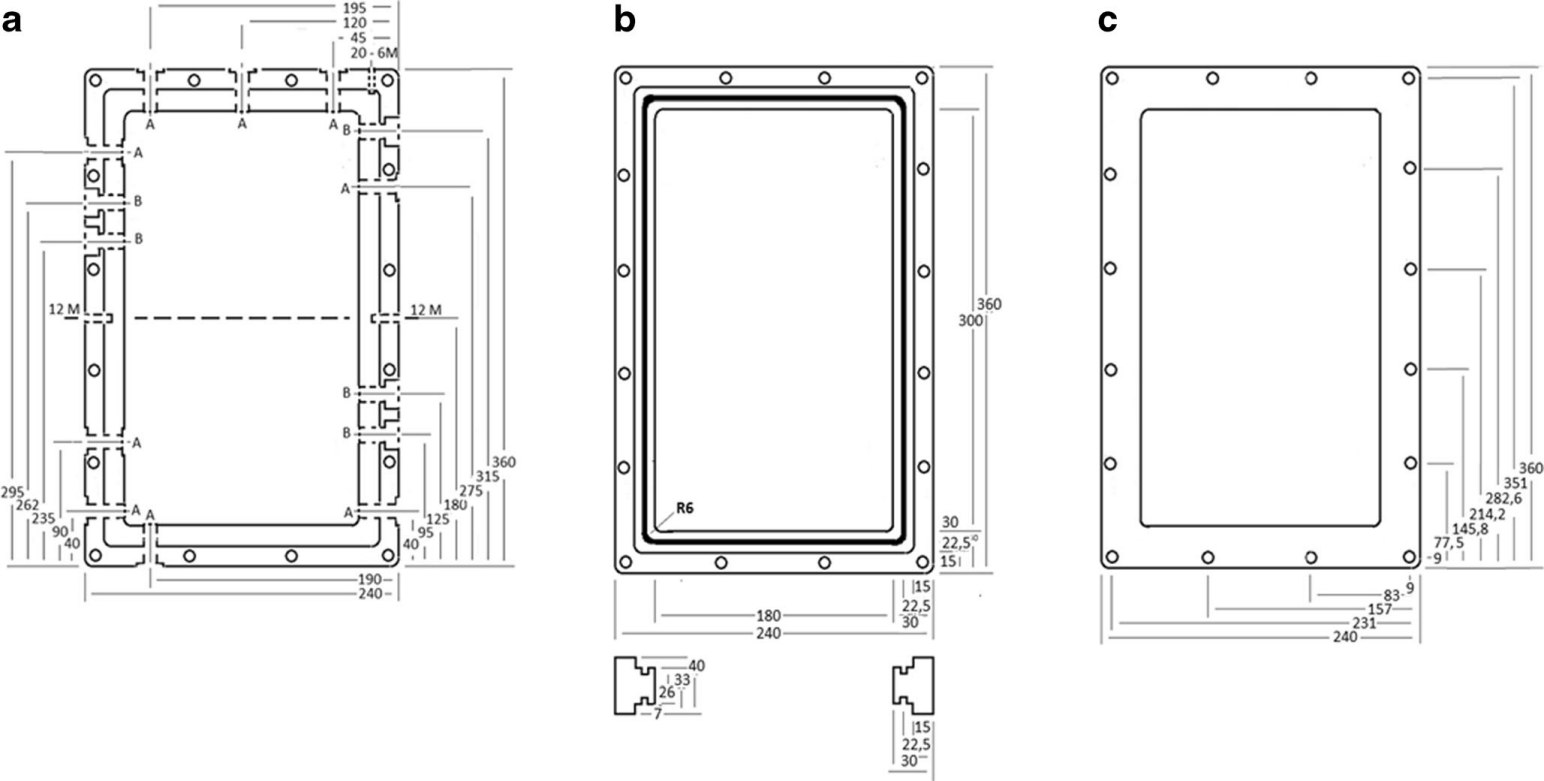
**a** Inner frame, showing the position of 14 holes with access to the culture chamber. The holes had two dimensions, ½” threads (*A*) and PG 13.5” (*B*). **b** Inner frame, showing dimensions of the frame and the tracks for the O-ring seal, and the tracks for the glass windows. **c** Outer frame, with position of the 16 holes used for holding the inner frame and two outer frames together

A large O-ring with the dimensions 313 × 7 mm made of Viton FKM 70 shore (Table 1, material 7) was placed in the dedicated track (Fig. 1b) of the inner frame (see detailed description below). The function of this O-ring was to seal the opening between the PFA-covered steel and the glass plate. Other connections were sealed with smaller O-rings made of Viton FKM 75 shore (Table 1, material 8). The O-rings have very low hydrogen penetration and are food-grade approved by the US Food and Drug Administration (FDA).

Septum size 12.5 × 3 mm of type GR-2 silicon rubber (Supelco) (Table 1, material 9) was used for all sampling ports in the bioreactor frame and in the gas collection unit. These septa were chosen for their low hydrogen leakage and self-sealing properties after needle penetration.

The ferrules of the Swagelok connections were in general made of stainless steel, with the exception of the ferrule used to connect the gas collection unit made of glass with the metal connection of the reactor. These ferrules were made of Teflon to avoid tension caused by contact between the glass and metal.

#### Glass

Glass windows of the bioreactor were made from fortified soda-lime-silica glass (Table 1, material 4), which proved resistant to autoclaving under the physical stress caused by the tension between the glass and the metal frame when mounted in the frame. Standard soda-lime-silica glass, commonly known as “window glass,” was proven inadequate due to a tendency to crack during autoclaving.

The gas collection unit and the gas exhaust tube were made from borosilicate glass (Table 1, materials 10 and 11). The units were autoclaved separately and mounted on the metal connection after autoclaving to avoid unnecessary tension.

#### Silicone peroxide tubes

Transparent and flexible silicone peroxide tubes were used in three places in the photobioreactor, i.e., the inlet tube for the air/CO_2_ mixture during bubbling phase of the cultivation, the inlet and outlet of the temperature regulation water circulating through the reactor chamber, and the outlet tube for water displacement attached to the gas collection unit in the hydrogen production phase (Table 1, material 12).

### Potential effects of materials on microalgae cultures

A small study was performed to evaluate the possible adverse effects of eight different materials on growth of microalgae cultures which could potentially be used in photobioreactor constructions.

The green microalga *Chlamydomonas reinhardtii* NIVA CHL153 (id. SAG 34.89) was cultivated in TAP medium (Harris 2009), with the modification that acetate was omitted and replaced by HCl for pH adjustment (TAP-ac). The effects of the following materials were tested: stainless steel ss316, stainless steel ss304, aluminium, copper (Cu), natural rubber, PVC, silicon peroxide, and polycarbonate (PC), in addition to a control without any added material. The samples were prepared by cutting each material into pieces with a surface area of 6 cm^2^. Each piece was thoroughly rinsed with deionized water and put into a 100-mL glass Erlenmeyer flask; 60-mL TAP-ac medium was added before the flasks were autoclaved. Three parallel flasks were prepared with each of the eight materials. Each flask was inoculated with *C. reinhardtii* to a density of 8 × 10^4^ cells mL^−1^ and OD_750_ of 0.005. The algae were cultivated on a shaking table at 120 rpm, the temperature was set to 29 °C, and the light intensity was 400 μmol photons m^−2^ s^−1^. Growth was followed by measuring OD_750_.

The algae cells were thereby exposed to the materials either by direct contact with the solid material in the bottle or in addition by any ions, monomers, or other constituents which could possibly have leaked out from the materials into the medium.

#### Results

**Fig. 2:**
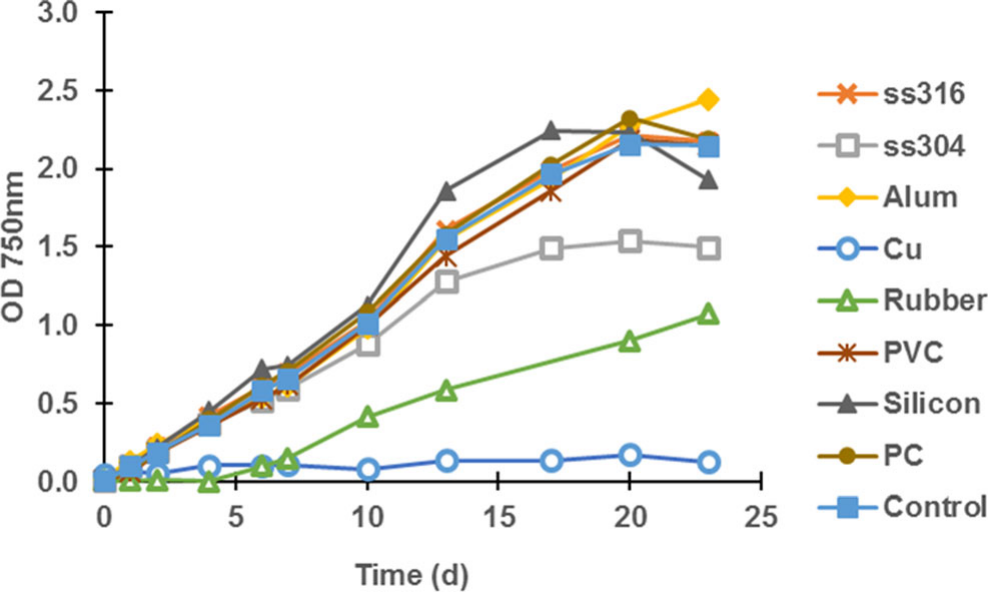
Effects of different materials on growth of *C. reinhardtii*. Materials with 6 cm^2^ surface area were added to the media before autoclaving, and each material was tested in triplicate cultures. The materials were stainless steel ss316, stainless steel ss304, aluminium, copper (Cu), natural rubber, PVC, silicon peroxide, and polycarbonate (PC), in addition to a control without any added material

As shown in Fig. 2, three of the materials appeared to have a clear effect on growth of the algae. The most significant effect was found in cultures exposed to Cu. All three parallel cultures exposed to a Cu cube showed the absence of growth. Cu^+^ ions in high concentrations have known toxic effects on microalgae (Debelius et al. 2009), and even if the algae cultures were prepared with solid metal cubes only, it is likely that metal ions may have detached and dissolved into the medium.

Another clear inhibiting effect was caused by natural rubber. After a lag phase of 4 days with no detectable growth, two of the parallel cultures started to recover. The third, however, did not survive. Natural rubber has previously been shown to have an inhibiting effect on algae (Skjånes et al. 2008; Williams and Robertson 1989); however, to our knowledge, this topic has not been extensively studied. Monomer units of the isoprene polymer, which natural rubber is composed of, have a known toxic effect (Melnick et al. 1994), and it is possible that leakage of this monomer into the medium caused the effect detected in this experiment.

The third material that showed an effect on the algae growth was the stainless steel alloy ss304. For the first 7 days of cultivation, its growth rate was similar to the control, but thereafter, the growth slowed down. The cultures reached stationary phase 3 days before the control. Stainless steel alloy ss304 contains high amounts of chromium (Cr) (18–20 %) and nickel (Ni) (8–10.5 %) that both have known toxic effects on algae (Jin et al. 1996; Singh and Rai 1991; Levy et al. 2008). It is possible that ions of these constituents leaked into the medium. Nutrient deprivation has previously shown to increase the inhibiting effect of metals on algae (Hall et al. 1989), a fact that could explain the late onset of the effect during incubation. However, one possible source of error in this particular case was that the shape of the metal was more rounded than the others, and moved around in the cultures at the shaking table. This could possibly have caused mechanical stress that inhibited the growth as the cultures became denser.

The results show that care must be taken in selecting materials for photobioreactor construction. This is also illustrated by the fact that all alloys of stainless steel contain metals with potentially toxic effects on algae in their ionic forms. Depending on the conditions such as the quality of the alloy, ions could escape from the metal and in theory affect the culture. Natural rubber should be avoided altogether. The aspects of the inhibiting effect of materials to be used in photobioreactor design should however be studied more extensively.

### Construction

#### Culture vessel

The culture vessel consisted of metal inner and outer frames with glass windows, a metal tube for temperature regulation, a metal tube for bubbling of the culture with air/CO_2_, and a total of 14 openings for sensors, sampling ports, culture inlet and outlet, gas exhaust, and gas collection units. Figures 1, 3, 4, 5, 6, 7, 8, 9, 12, 13, and 14 illustrate the design of the culture vessel part of the photobioreactor.

**Fig. 3:**
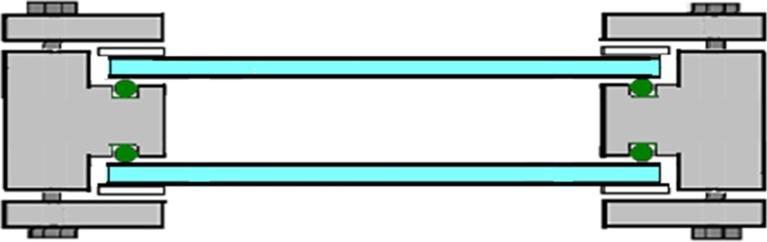
Cross section of the culture vessel of the photobioreactor. The inner and the outer frames are held together by 16 bolts, and the two glass plates on each side of the frame are protected from contact with the aluminium outer frames with 1-mm silicon gaskets. An O-ring forms a hydrogen tight seal between the glass plates and the inner steel frame. The edge of the glass plates have a 3-mm distance from the inner frame, to allow for the unavoidable movement of the materials during autoclaving, without creating tension in the glass

**Fig. 4:**
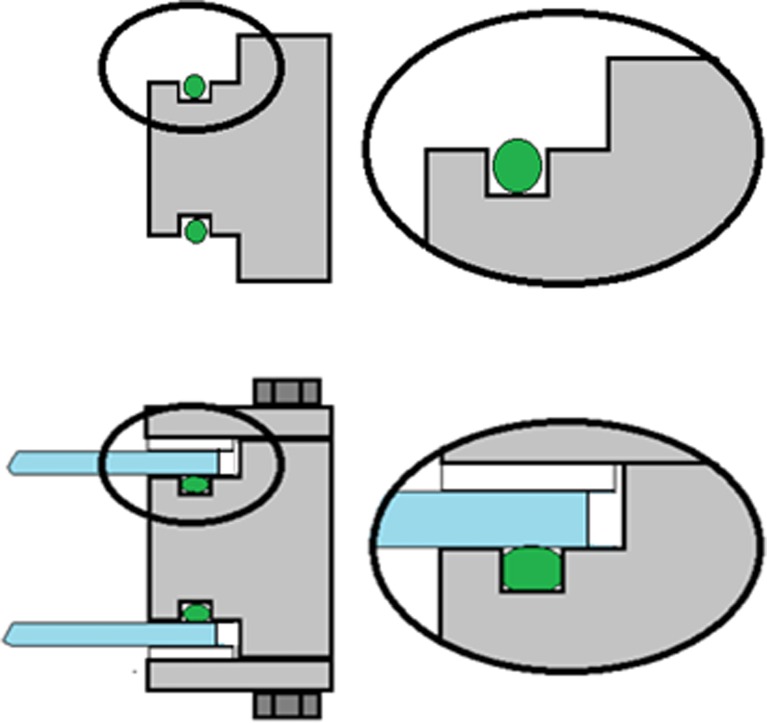
The FKM O-ring forming the seal between the glass plates and the inner frame was placed inside a track designed to compress the O-ring sufficiently to form a hydrogen tight seal, without breaking the polymer bonds of the material

**Fig. 5:**
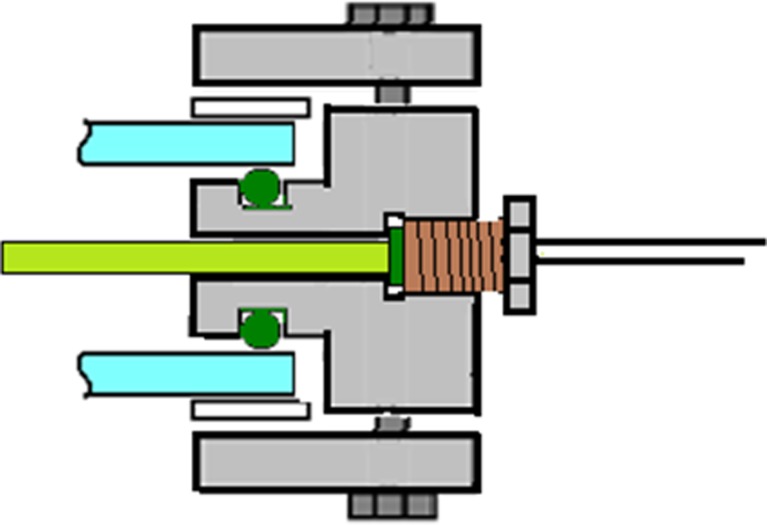
Sensor insertions. Cross section of a sensor inserted through one of the “B” holes with ½” threads of the inner frame. The opening is sealed with an FKM O-ring below the threaded part of the hole

**Fig. 6:**
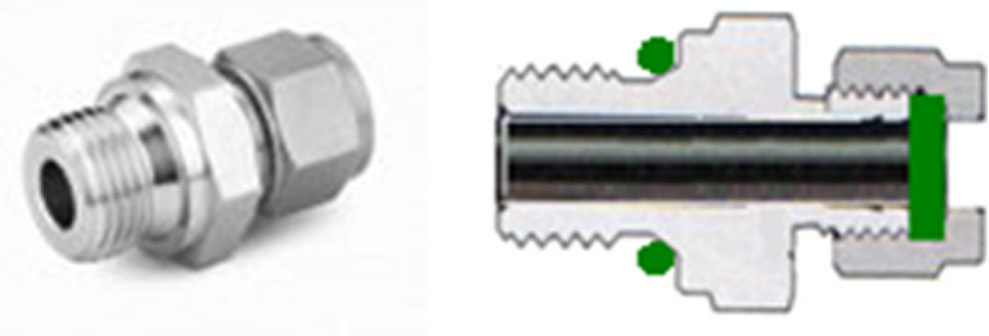
Sampling port, made from a standard Swagelok male tube fitting, where the locking cones were replaced by a GR-2 septa

**Fig. 7:**
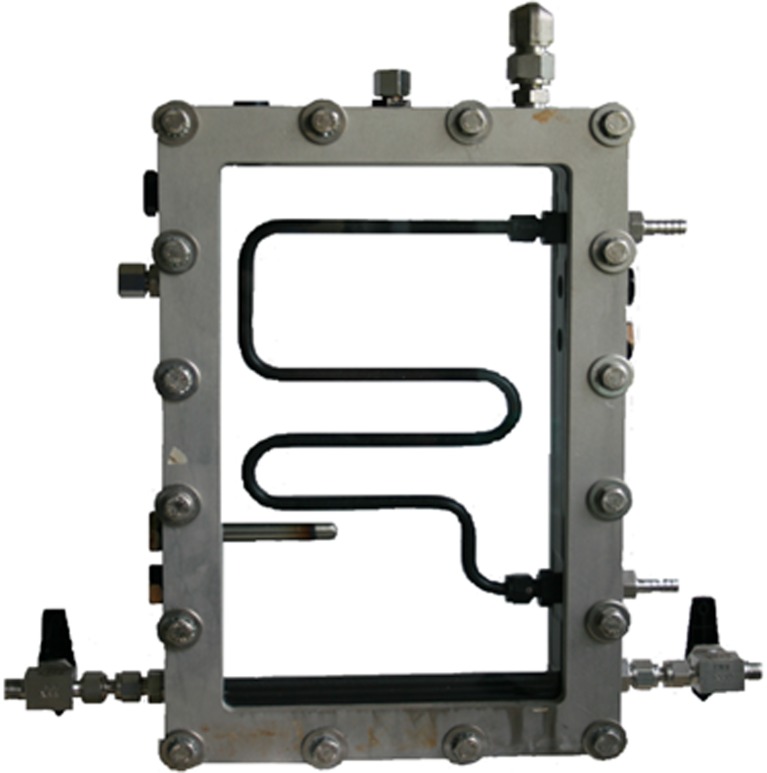
The assembled frames, showing the tube for temperature regulation inside the culture chamber. The temperature regulation tube was shaped to ensure that any conflict with inserted sensors was avoided. The gas inlet tube with valves can be seen at the bottom

**Fig. 8:**
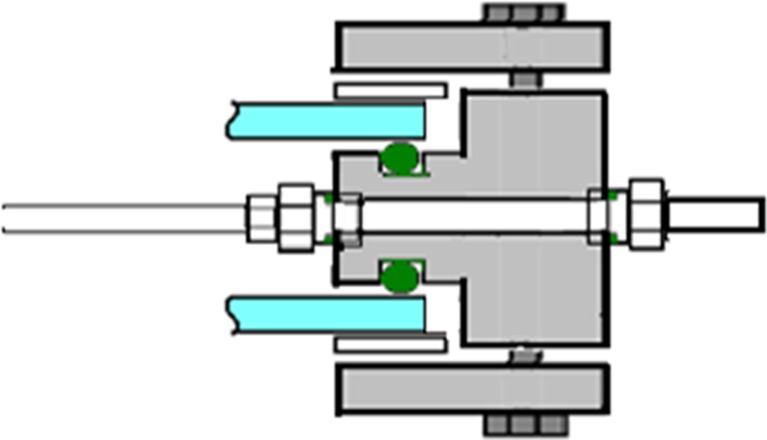
Temperature regulation. Cross section of the reactor frame, showing the tube for temperature regulation attached to the inside of the inner frame with Swagelok fittings. On the outside of the inner frame, a second fitting provided an adapter for attaching a silicon tube for circulation of water

**Fig. 9:**
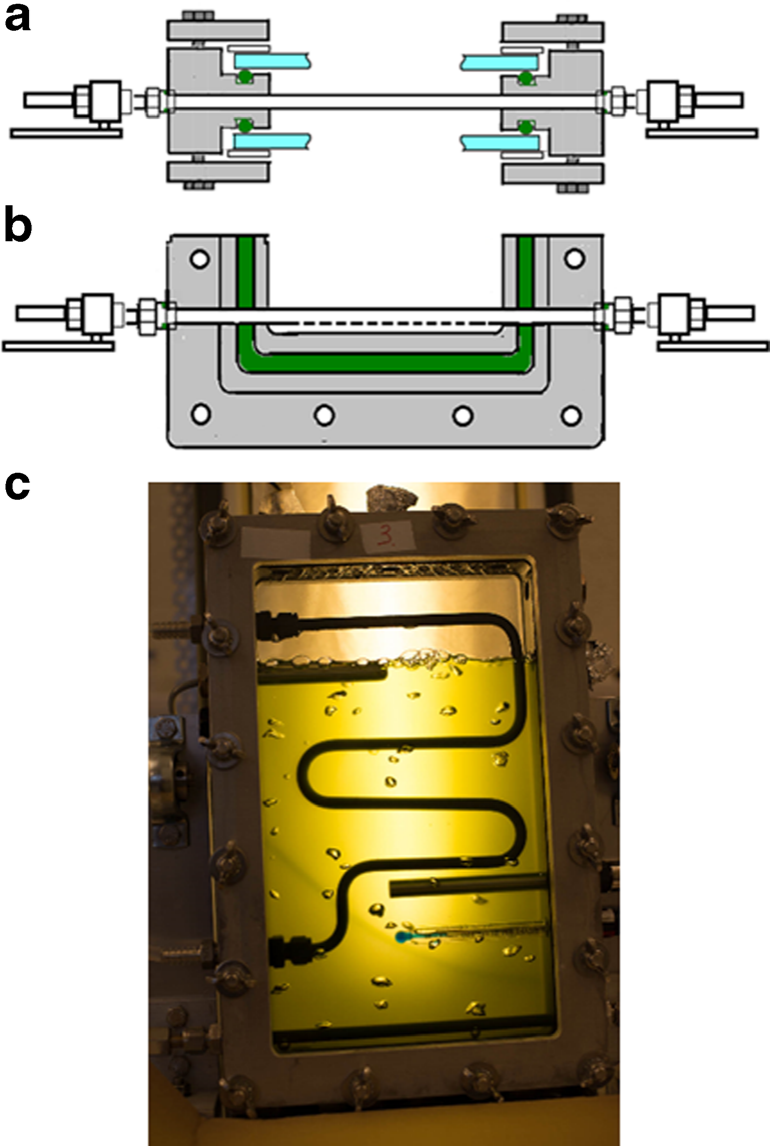
Gas inlet tube. **a** Cross section of the reactor frame, showing the gas inlet tube attached to the inner frame with Swagelok fittings on the outside of the frame. A ball valve for controlling the gas inlet was connected to the tube on each side. **b** Side view of the inner frame, showing the 1-mm openings for sparging on the bottom side of the gas inlet tube. **c** Culture sparged with air/CO_2_

The volume of the culture vessel part of the photobioreactor was selected to be about 1.6 L, which was estimated to be an appropriate volume for our purpose. The chosen light path was 30 mm. The outer measurements of the vessel were 240 × 360 × 40 mm (*W* × *H* × *D*), and the respective inner measurements of the culture compartment were 180 × 300 × 30 mm, resulting in a culture chamber volume of 1.62 L.

Figure 3 shows an overview with a cross section of the culture vessel frames assembly. The inner and outer frames were held together by 16 bolts. The two glass plates on each side of the frame were protected from contact with the aluminum outer frames with 1-mm silicon gaskets. An O-ring formed a hydrogen tight seal between the glass plates and the inner steel frame.

The stainless steel inner frame had 15 × 7 mm (*W* × *D*) tracks on the inner side of the frame, for placing the glass windows on each side of the frame (Fig. 1b). Inside this track, another 9.5 × 5.8 mm (*W* × *D*) track was placed. This smaller track fit the 313 × 7-mm O-ring that formed a gas-tight seal between the inner frame and the glass windows. The size of the track was estimated to give a 17 % radial compression of the O-ring, considered to give an optimal gas-tight seal (Fig. 4).

The glass windows measured 204 × 324 × 6 mm (*W* × *H* × *D*), allowing a 3-mm space between the glass and the metal of the inner frame when the glass was placed inside the track described above. A 10 × 1- mm (*W* × *H*) silicon rubber gasket was placed on top of the glass in order to form a space between the glass and the metal of the outer frame (Fig. 3). The two aluminium outer frames placed on top of the rubber gaskets measured 240 × 360 × 10 mm (*W* × *H* × *D*) with a 182 × 300 - mm opening (Fig. 1c).

The inner and outer frames had 16 holes where 90-mm-long steel bolts connected the frames (Figs. 1c and 3). For a hydrogen tight seal, the screws were tightened to a torque of 42 Nm. This design resulted in an even pressure between the glass and the O-rings securing thereby a hydrogen-tight seal, without creating tension in the glass. The design allowed for the unavoidably uneven movements of the steel, aluminium, and glass caused by autoclaving, without loss of the hydrogen-tight properties of the culture vessel part of the bioreactor.

The inner frame had 14 holes allowing access to the culture chamber (Fig. 1a). Five of the holes (marked “B”) had PG 13.5 threads, which is a standard thread size used for pH and dissolved oxygen sensors. See Fig. 5 for installation of the sensors. However, this size threads had a very poor selection of other off-the-shelf parts. Therefore, the remaining nine holes (marked “A”) had ½” threads, a standard size where there is a great selection of bolts, fittings, and connections useful for different photobioreactor setups. The inner frame also had a 12M hole on each side, and a 6M hole on the top, for mounting the culture vessel on the stand (see below). Sampling ports were made from standard male tube connectors fitting holes “A,” where the locking cones were replaced by a GR-2 septa (Supelco) (Fig. 6). The number and positions of these sampling ports were determined for each experimental setup.

Temperature regulation of the culture was implemented with a temperature-regulated Teflon-covered stainless steel tube of 8 mm diameter. This tube was positioned inside the culture compartment (Fig. 7) and used for circulating water for heating or cooling. The length and shape of the tube were designed to avoid interaction between the temperature tube and the sensors, allowing space for all five sensor openings to be used (Fig. 1a, holes “B”). The inlet and outlet of the tube were connected to the inner frame through two of the holes “A” shown in Fig. 1a. On the outside of the frame, a tube adapter for connecting a silicon tube was inserted. O-ring seals were used both on the inside and the outside of the hole. The temperature tube connection design is shown in Fig. 8. Swagelok fittings with two cone rings were used for all connections, as other fittings proved to result in hydrogen leakage.

For the addition of the air/CO_2_ mixture, alternatively other gases such as N_2_, an 8-mm diameter Teflon-covered steel tube was installed close to the bottom of the culture chamber, through two of the “A” openings shown in Fig. 1a. The design of the gas tube is shown in Fig. 9. The tube was attached to the reactor frame with Swagelok fittings, with an O-ring seal. On the bottom side of the tube, six holes of 1-mm diameter were made for injecting air/CO_2_ to make evenly distributed bubbles, creating agitation and gas exchange. On both ends of the tube, a ball valve was installed for simple start and halt of air/CO_2_ addition. On the other side of the valve, a tube adapter was connected.

#### Reactor stand/ rocking motion

**Fig. 10:**
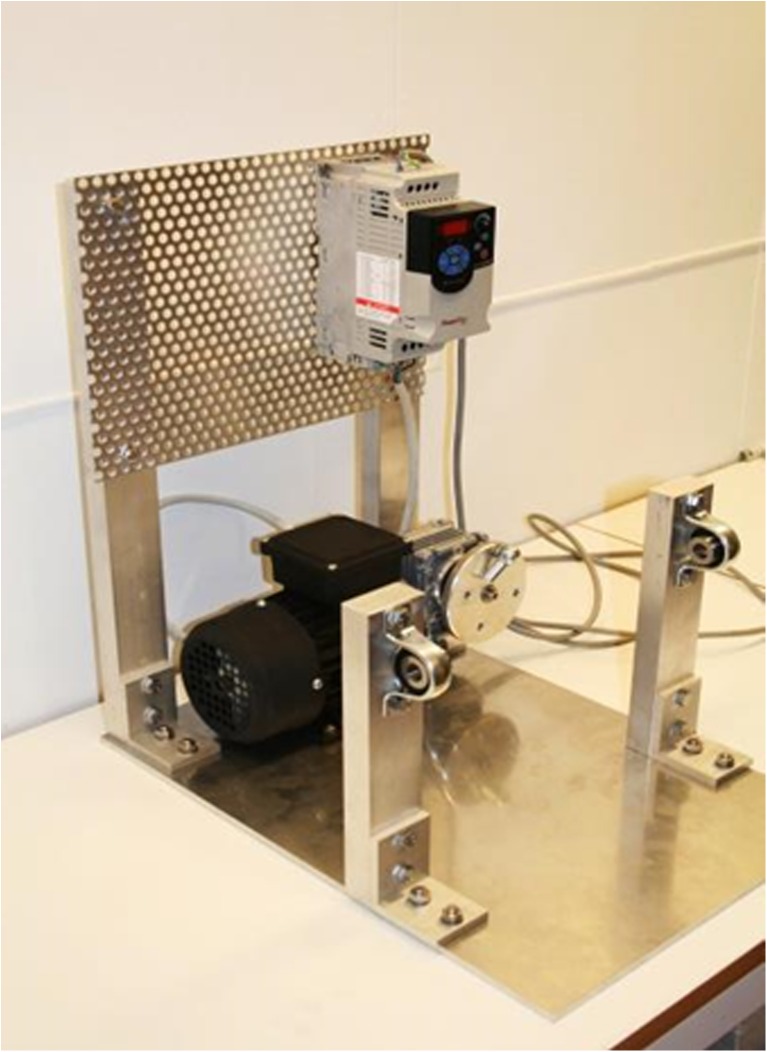
Photobioreactor stand, including motor, rotating disc, transmission, frequency converter, and holders for the culture vessel

A reactor stand (Fig. 10) was constructed of aluminium, consisting of a bottom plate 350 × 500 mm (*W* × *D*), with two 500-mm tall aluminium rods at the back end of the plate. The rods were attached with aluminium brackets and stainless steel screws. Between the two rods, a mounting plate was attached for mounting the frequency converter and other instruments. The motor and transmission were mounted on the bottom plate between the aluminium rods. Also on the bottom plate, 165 mm from the front, two 250-mm tall aluminium rods were mounted for supporting the bioreactor tilt, with bearing housings on the top. A 12-mm bolt was inserted through the bearing housing on each side and screwed into the reactor frame. This construction was holding the culture vessel and made it possible to easily tip the bioreactor from the vertical position to the horizontal position, and vice versa.

**Fig. 11:**
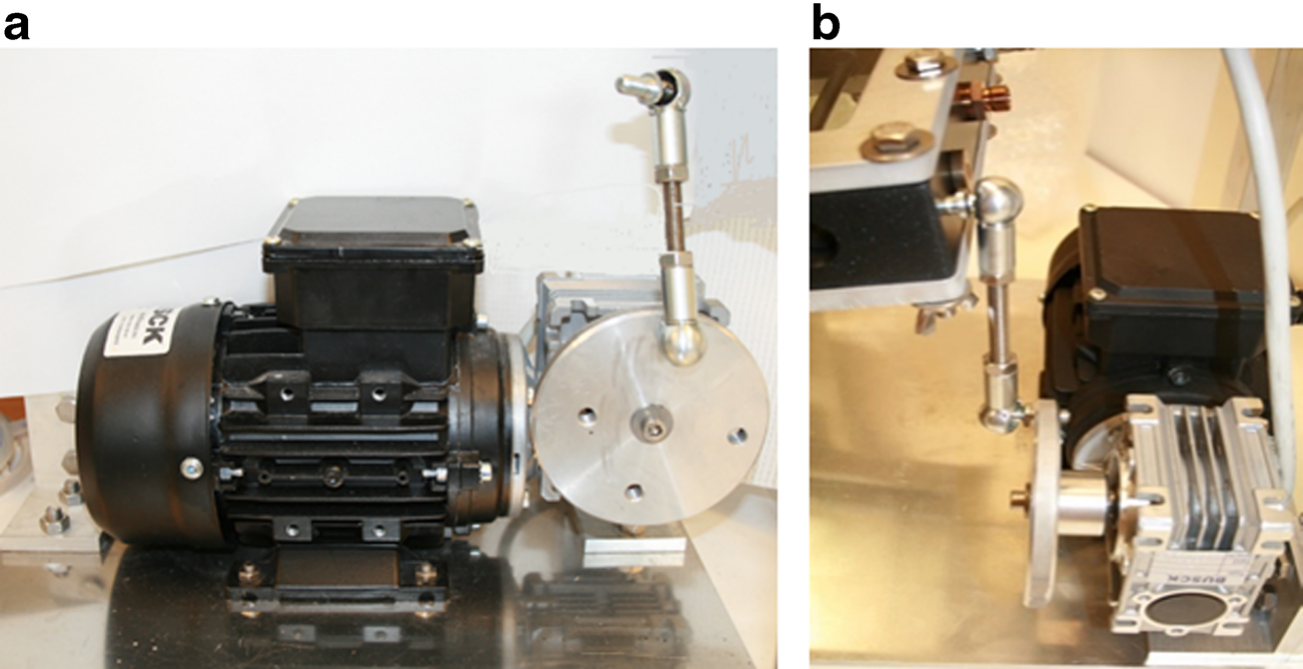
Motor for rocking motion. **a** The motor with a rotating disc for attaching the reactor frame to the motor. The disc had four holes with different positions, which could be used to select the angle of the rocking motion. **b** The frame of the culture vessel attached to the motor, for creating the rocking motion

To form the rocking motion during the hydrogen production phase, a motor and a transmission were placed on the back end of the bottom plate (Figs. 10 and 11). The motor had a 230/400 voltage, power of 0.25 kW, and transmission ratio of 7.5:1. This resulted in a velocity reduction from 1350 to 180 rpm to the disc creating the rocking motion of the bioreactor. A power-flex frequency converter reduced the motion of the bioreactor further. At maximum frequency of 60 Hz, the disc rotated 35 rpm, and at a minimum frequency of 1 Hz, the disc rotated 1 rpm. The photobioreactor was run normally at a frequency of 16.5 Hz, with a rotation of 9 rpm.

The rotating disc had four holes with different distances from the center. This affected the tilting angle during the rocking motion of the photobioreactor. When the transmission arm was attached to the hole converged toward the center, the reactor’s tilting movement was small. Likewise, when the transmission arm was attached to the hole far from the center, its tilting movement was larger. The required movement of the alga culture was obtained by combining the motion with the frequency of the frequency converter. Figure 11b shows the bioreactor connected to the motor system for rocking motion.

#### Gas collection

**Fig. 12:**
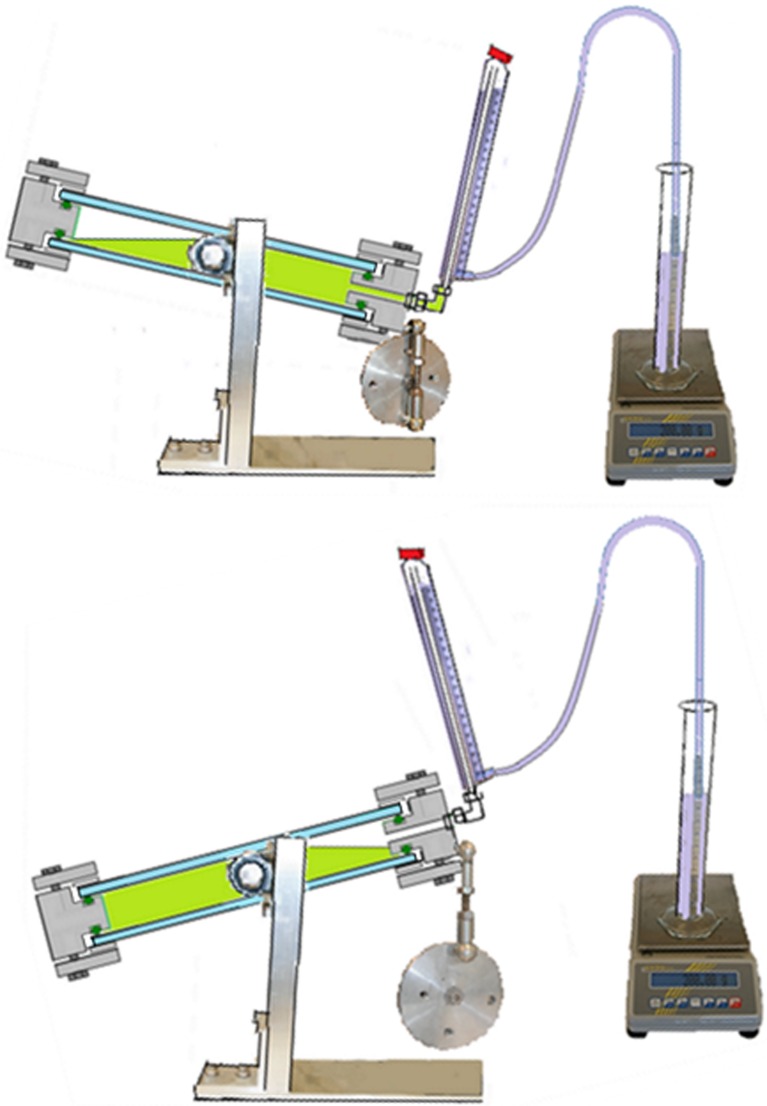
Gas collection system. A custom-made glass tube with an inner and outer compartment was used to measure the volume of produced gas by water replacement. During the rocking motion, the culture was able to run freely in and out of the bottom part of the collection tube, meaning that when the gas collection side of the culture vessel was in the upper position, produced gas could escape into the collection tube. The volume of produced gas could be recorded manually using the scale on the collection tube or the scale of the glass cylinder placed on the balance, or logging the signal from the balance connected to the logging system. The gas collection unit had a septum for gas sampling, to allow for hydrogen % measurements from the collected gas in addition to the hydrogen measurements from the culture vessel headspace

Hydrogen gas produced in the culture vessel was captured by a gas collection unit made of glass (Fig. 12). The unit consists of an outer glass tube filled with water and an inner glass tube where the lower part was connected to the culture vessel through a steel Swagelok connection mounted in a 90° angle. The glass tube was fastened to the steel connection with a Teflon ferrule to avoid tension between the steel and the glass. The opening at the bottom of the steel connection measured 8 mm diameter, which was wide enough for the culture to run freely in and out of the opening during the rocking motion. This means that when the hydrogen collection side of the culture vessel was in the upper position, the gas could pass freely into the collection unit. When smaller diameters of this lower opening were used, the culture was sometimes left inside the connection and consequently pushed out into the gas collection unit during hydrogen production.

An opening at the bottom of the outer glass tube with a tube adapter was attached to a silicon tube inserted into a glass cylinder filled with water, placed on a balance. As the outer glass tube filled with gas from the bioreactor, the water was replaced and moved to the cylinder, and the amount of water was recorded. Due to the low solubility of hydrogen in water, this design functioned as a water trap. The potential problem of the high permeability of silicon peroxide to hydrogen was avoided by assuring that there was a water trap present between the silicon tube and the gas phase in the collection unit. The top of the collection unit contained a screw cap with a septum for sample extraction. This design allowed for a stable connection between the culture vessel and the gas collection unit, minimizing the risk of leakage caused by the rocking motion. The glass collection unit was designed in four different sizes, selected for each experiment based on the expected amount of hydrogen produced.

The gas collection system allowed for three ways of quantifying hydrogen production: Larger volumes of hydrogen were quantified as volume of gas captured in the gas collection unit and/or measured as water replacement registered by a balance attached to the logging unit. Detection of any volume of hydrogen was always combined with measurement of hydrogen % in the gas. Amounts of hydrogen too low to be measured as a significant volume of hydrogen emitted from the reactor were most accurately detected as hydrogen % in the headspace of the reactor. Samples of the headspace gas could be extracted manually using a syringe or with an automated gas sampling system. The gas composition could be measured with a GC, HPLC, MS, or any other suitable method.

#### Photobioreactor setup

**Fig. 13:**
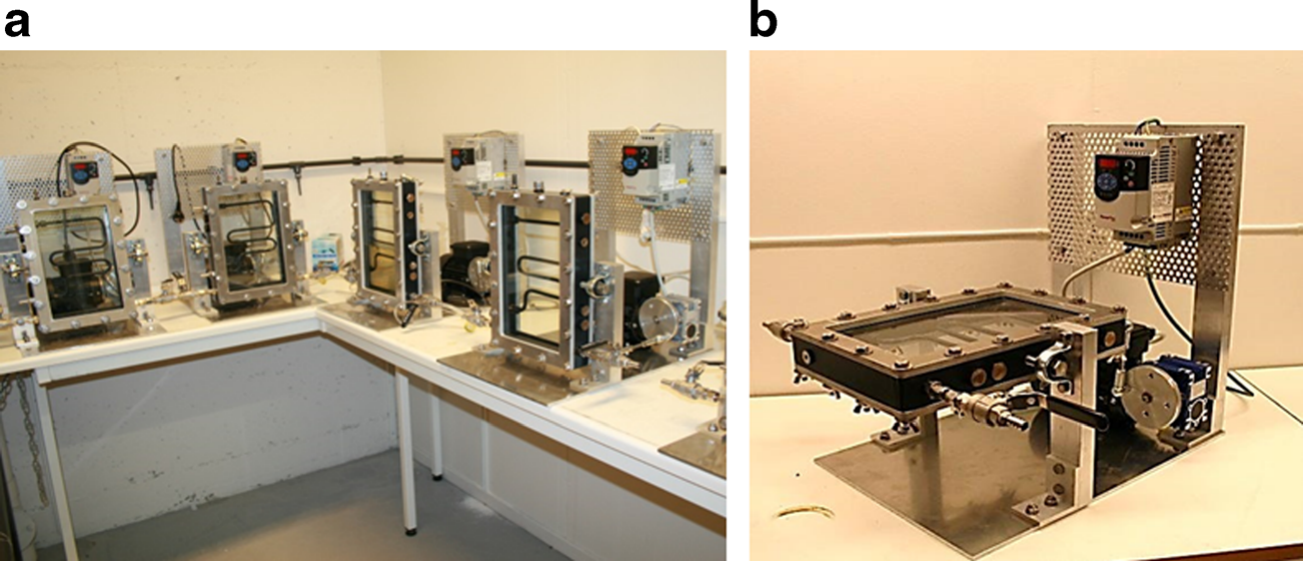
Assembled photobioreactors. **a** Photobioreactors with the culture vessel in vertical position for algae cultivation. **b** Photobioreactor with the culture vessel in horizontal position, for hydrogen production

**Fig. 14:**
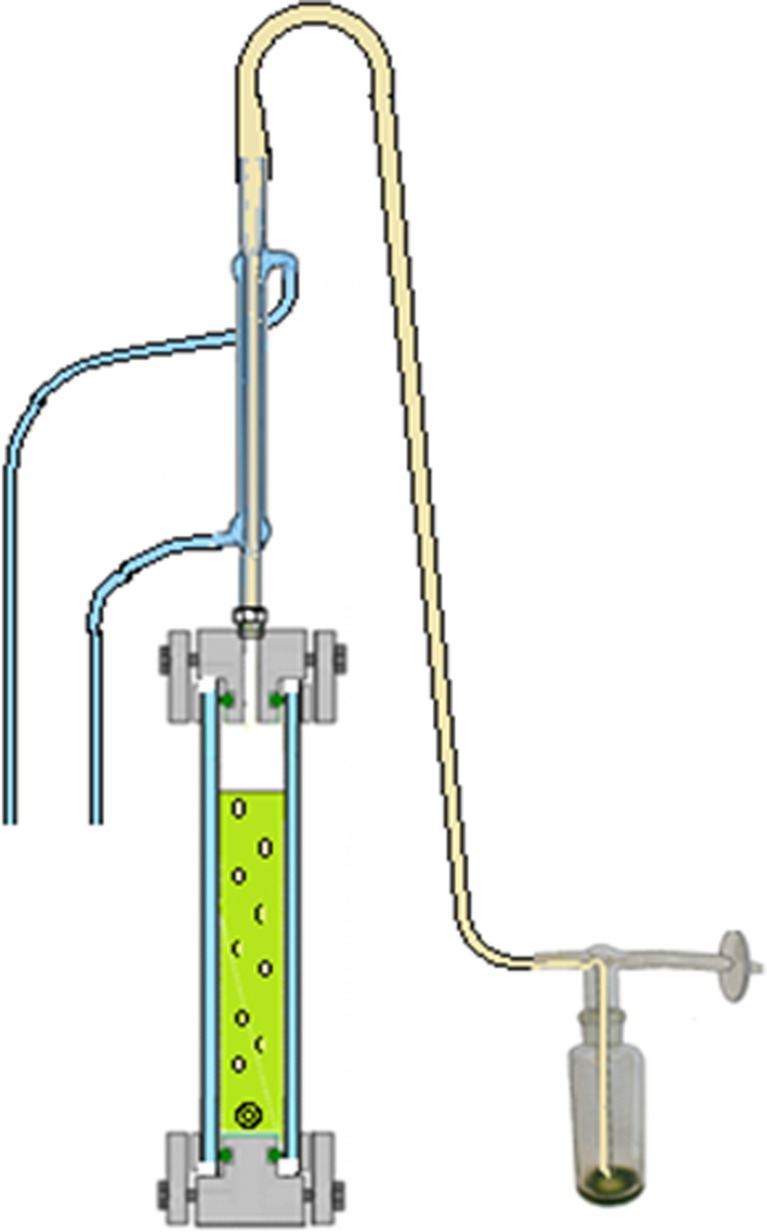
Air exhaust system. The exhaust air was cooled down with a water jacket circulating cold water. A foam catching bottle was attached to the exhaust tube

When the reactor was used for algae cultivation, the culture vessel was placed in a vertical position, as described above and shown in Fig. 13a. The cultivation setup included injecting a mixture of air/2–5 % CO_2_ through the air bubbling tube illustrated in Fig. 9, connected using a silicon tube through a sterile filter. The exhaust air was evacuated through a glass tube on the top of the bioreactor, which was cooled down by circulating cold water around the outside of the tube to condensate any evaporation. In cases where foam was formed on top of the cultures, a foam collection flask was attached to the exhaust evacuation tube. The air exhaust system is shown in Fig. 14. Sampling ports for culture extraction during the experiments were placed on the top and on the side, where culture samples could easily be removed with a syringe. Light panels were placed vertically on the bioreactor stand between the reactor vessel and back panel, or in front of the reactor. The light panels were not attached to the stand, this in order to allow easy adjustment of distances as a part of light intensity alterations. In the initial stages of the hydrogen production phase, the reactor was kept in the vertical position, with injection of N_2_ instead of air to secure anaerobic conditions and avoid the addition of O_2_.

During hydrogen production, the culture vessel was placed in a horizontal position as shown in Fig. 13b. Sampling ports were placed on the side and at the bottom of the culture vessel, for extracting both culture samples and gas samples from the headspace. The gas collection system described above was installed and the culture was agitated by using the rocking motion function. Since the pressure inside the culture chamber was an important factor for measuring the volume of produced gas, culture sample removal was performed with care. Two syringes were used in parallel: one syringe was used to extract the culture sample, and the other syringe was filled with N_2_ which was injected into the culture chamber through a sterile filter at the exact same speed. The water level in the gas collection tube was used as a measure to ensure that the extracted culture volume was replaced by an equivalent volume of N_2_. Light panels were placed in an independent steel rack horizontally above the culture chamber.

#### Hydrogen collection—sources of error

It is clear that certain materials commonly used in other types of photobioreactors are hydrogen permeable. Through our work, we have experienced that connections easily become weak points where the risk of hydrogen leakage is particularly high. All materials used in the photobioreactor described herein were carefully selected to prevent leakage of hydrogen gas, as described above.

The following procedure for detecting potential leakage of hydrogen from the reactor vessel was performed before each experiment: The reactor vessel was filled with hydrogen to 0.6–1 bar overpressure. The reactor was left for 0.5–1 h to stabilize, and all connections and seals were carefully examined using a hydrogen “sniffer” of type GM 5 (Schütz, Germany). The instrument was set to absorb 1.2 L gas min^−1^, and a detection of hydrogen <4 ppm was recorded as acceptable. In addition, the overpressure in the reactor vessel was measured using a PBT pressure transmitter (SICK AG, Germany) and logged overnight. Any detectable reduction in pressure was recorded as unacceptable and led to appropriate improvements such as tightening of screws or replacements of gaskets.

**Fig. 15:**
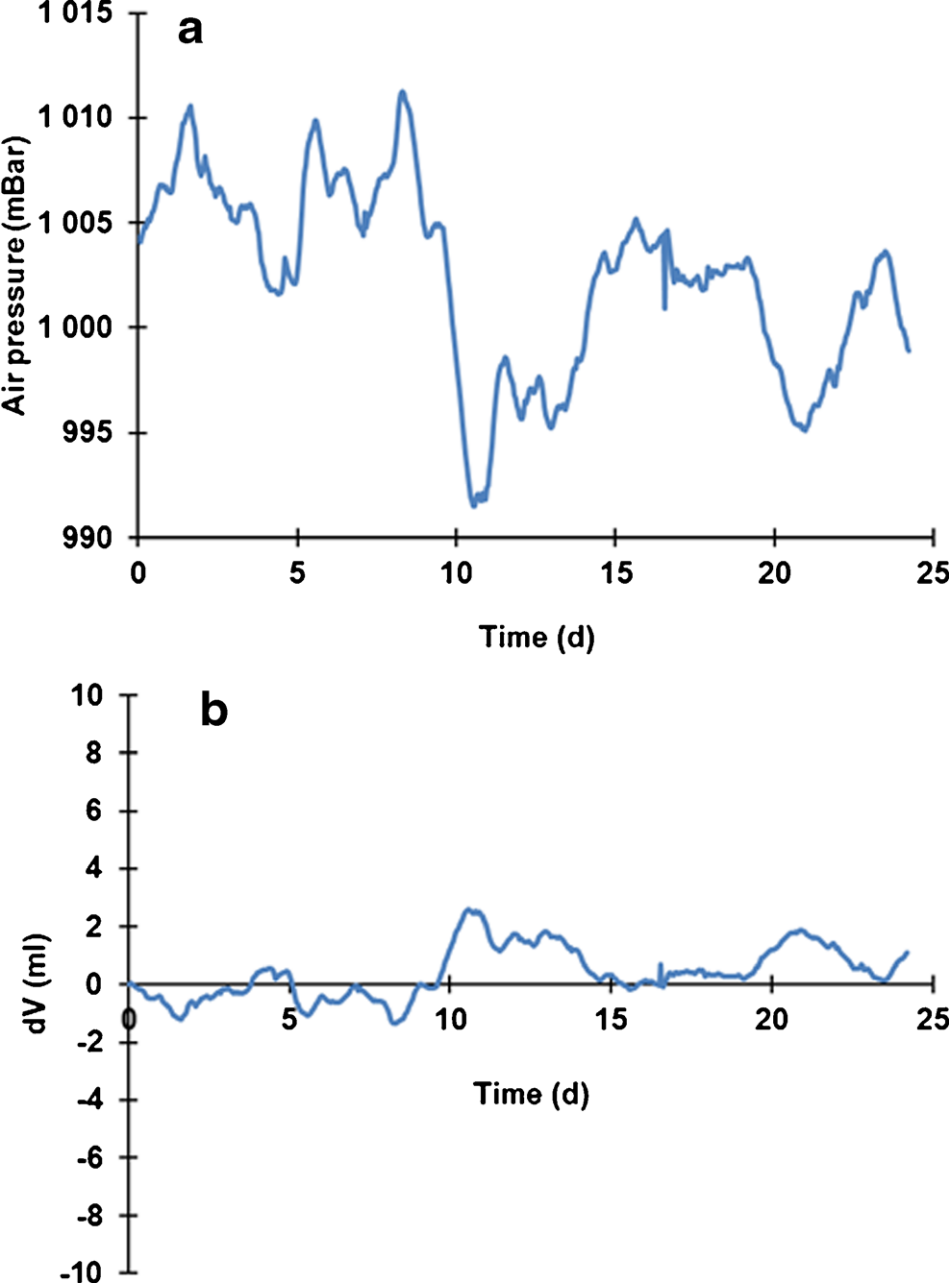
Air pressure and potential errors in the gas production measurement system. **a** Example of the variation in air pressure at Ås, Norway, in a given period. **b** The theoretical consequence of the air pressure for the volume of a 200-mL bioreactor headspace

The measurement system for gas production volume proved to be sensitive to changes in external air pressure, but only to a limited extent. Large changes in external air pressure during the gas collection phase of the experiments led to small errors in the detected volume of gas produced. An example of such inaccuracy is given in Fig. 15. The theoretical changes in volume was calculated according to the equation *p*_1_ × *V*_1_ = *p*_2_ × *V*_2_. This example was based on the air pressure at Ås, Norway (meteorological data supplied by FAGKLIM, NMBU), through a given experimental period of 24 days, in a photobioreactor where the headspace was 200 mL. For this given period, the maximum variation in calculated volume was ±2 mL. This variation in air pressure was found to be fairly representative for the variation throughout the year.

### Control system

**Fig. 16:**
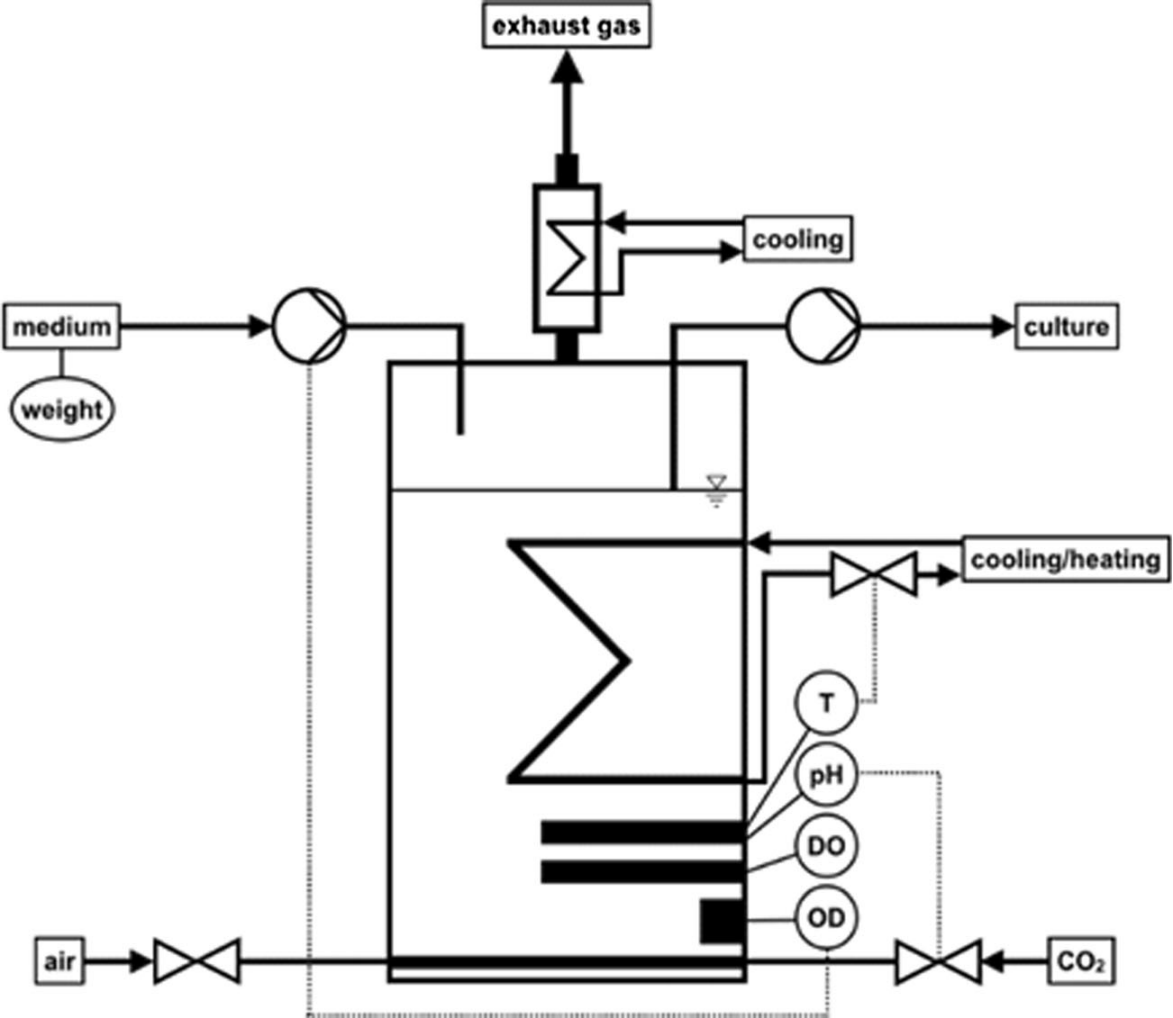
Control system of the flat panel photobioreactor system. *T* temperature, *DO* dissolved oxygen, *OD* optical density

**Fig. 17:**
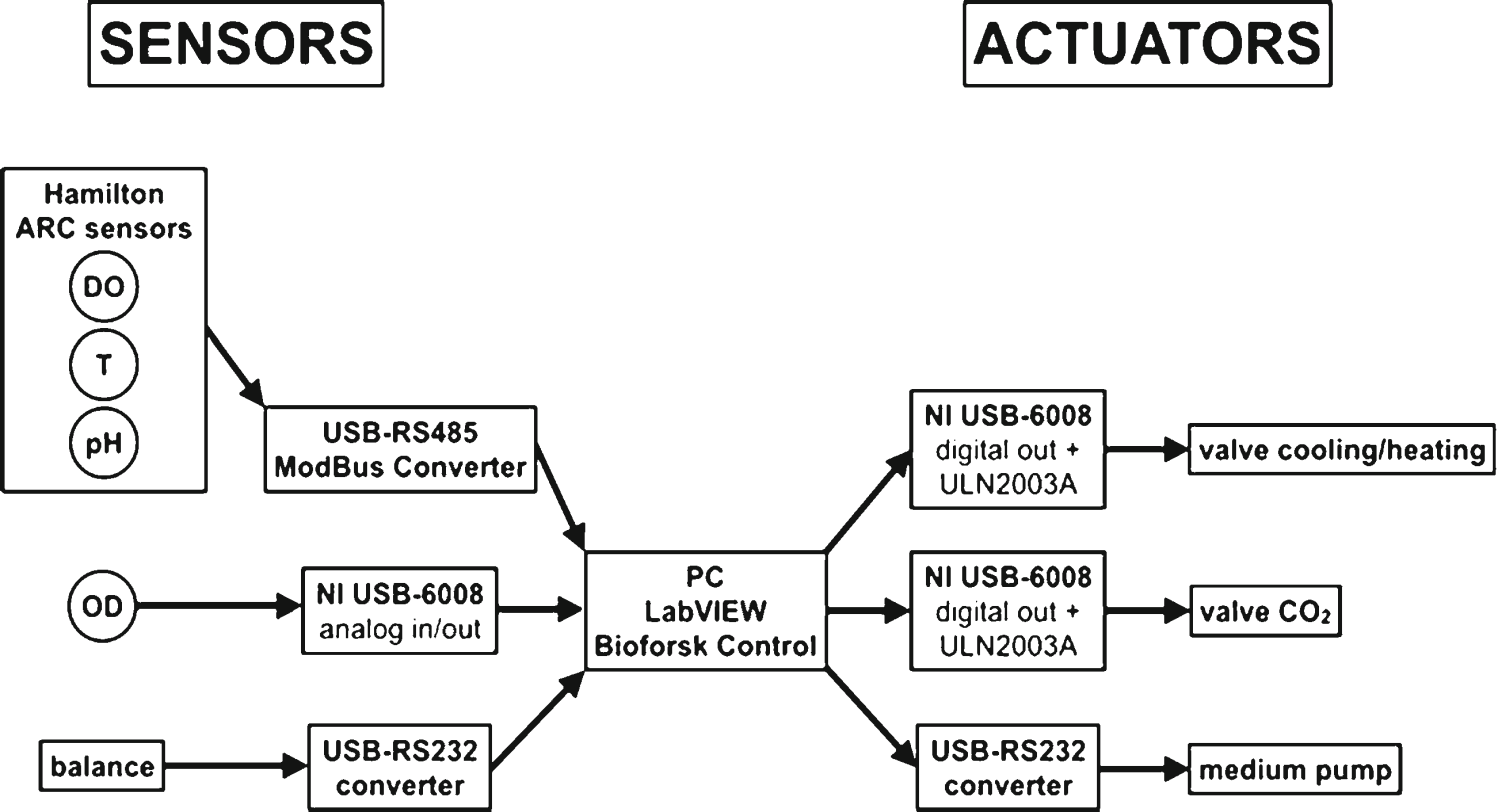
Implemented sensors and actuators. *T* temperature, *DO* dissolved oxygen, *OD* optical density

The bioreactor was controlled by the custom-made control software “Bioforsk Control” that was based on the graphical programming language “LabVIEW” (National Instruments, USA). An overview of the control system is sketched in Fig. 16. The sensors and actuators discussed below were implemented (Fig. 17 and Table 2).

**Table 2:** Implemented sensors and actuators of the control system

Parameter	Sensor/actuator	Connection to PC
Temperature	Easyferm Plus Arc, Hamilton Company, USA	USB-Nano-485, CTI GmbH, Germany
pH	Easyferm Plus Arc, Hamilton Company, USA	USB-Nano-485, CTI GmbH, Germany
Dissolved oxygen (DO)	Visiferm DO Arc, Hamilton Company, USA	USB-Nano-485, CTI GmbH, Germany
Turbidity/optical density (OD)	IR-LED (890 nm), TSHF6210, Vishay, USA + photodiode (940 nm) with integrated amplifier, TSL260R-LF, AMS-TAOS USA Inc., USA	USB-6008 AD-converter National Instruments, USA
Weight of hydrogen displaced water or feed bottle	Kern 572, Kern & Sohn GmbH, Germany	USB-RS232 converter, FTDI, UK
Control of cooling/heating water	Valve VDW31, SMC Corporation, Japan	USB-6008 AD-converter National Instruments, USA + Darlington transistor array ULN2003A, Texas Instruments, USA
Control of CO_2_ supply	Valve VDW21, SMC Corporation, Japan	USB-6008 AD-converter National Instruments, USA + Darlington transistor array ULN2003A, Texas Instruments, USA
Control of medium flow	Peristaltic pump Ismatec Reglo Digital, IDEX Health & Science GmbH, Germany	USB-RS232 converter, FTDI, UK

#### Sensors for temperature, pH, and dissolved oxygen

For the measurement of the temperature, the pH, and the DO concentration, the autoclavable sensors “Easyferm Plus Arc” (pH and temperature) and “Visiferm DO Arc” (DO) were used (both from Hamilton Company, Switzerland). Supplied with 24 V, the sensors were connected to the computer system via a USB-RS485-Modbus converter (USB-Nano-485, CTI GmbH, Germany). Drivers for LabVIEW were supplied by the manufacturer.

#### Turbidity/optical density sensor

For the measurement of OD, an IR-LED (890 nm, TSHF6210, Vishay, USA) and a photodiode with an integrated amplifier (940 nm, TSL260R-LF, AMS-TAOS USA Inc., USA) were mounted on both sides of the bioreactor glass windows (optical path = 4 cm). To adjust the dynamic range to the biomass concentration, the voltage of the LED could be changed by using an analog output channel (0–5 V) of a USB-6008 AD-converter (National Instruments, USA). Typically, the voltage was varied in a range between 1.3 (low biomass) and 2 V (high biomass). The analog voltage signal (0–5 V) of the photodiode was converted to a digital signal by an analog input channel of a USB-6008 AD-converter (National Instruments, USA).

#### Balance

For monitoring the amount of hydrogen produced (displaced water) or medium pumped into the bioreactor in continuous mode (chemostat, turbidostat), the weight was measured by a balance Kern 572 (Kern & Sohn GmbH, Germany). The balance was connected to the computer system via a USB-RS232 converter (FTDI, UK). Drivers for LabVIEW were supplied by the manufacturer.

#### Valves for cooling/heating water and CO_2_ supply

Based on the temperature signal, the temperature in the bioreactor was regulated by a proportional controller, which was switching a valve (VDW31, SMC Corporation, Japan), for controlling the flow of cooling or heating water through the heat exchanger in the bioreactor. Similarly, the pH was regulated by switching a valve (VDW21, SMC Corporation, Japan) in the CO_2_ supply line. The digital signal from the computer system was converted to a 24-V supply voltage by a digital output channel of a USB-6008 AD-converter (National Instruments, USA) in connection with a Darlington transistor array ULN2003A (Texas Instruments, USA).

#### Medium pump

In continuous mode, the medium was pumped with either a set flow rate (chemostat) or by a changing flow rate based on the OD signal (turbidostat) with a peristaltic pump Ismatec Reglo Digital (IDEX Health & Science GmbH, Germany). The pump was connected to the computer system by a USB-RS232 converter (FTDI, UK). Drivers for LabVIEW were supplied by the manufacturer.

**Fig. 18:**
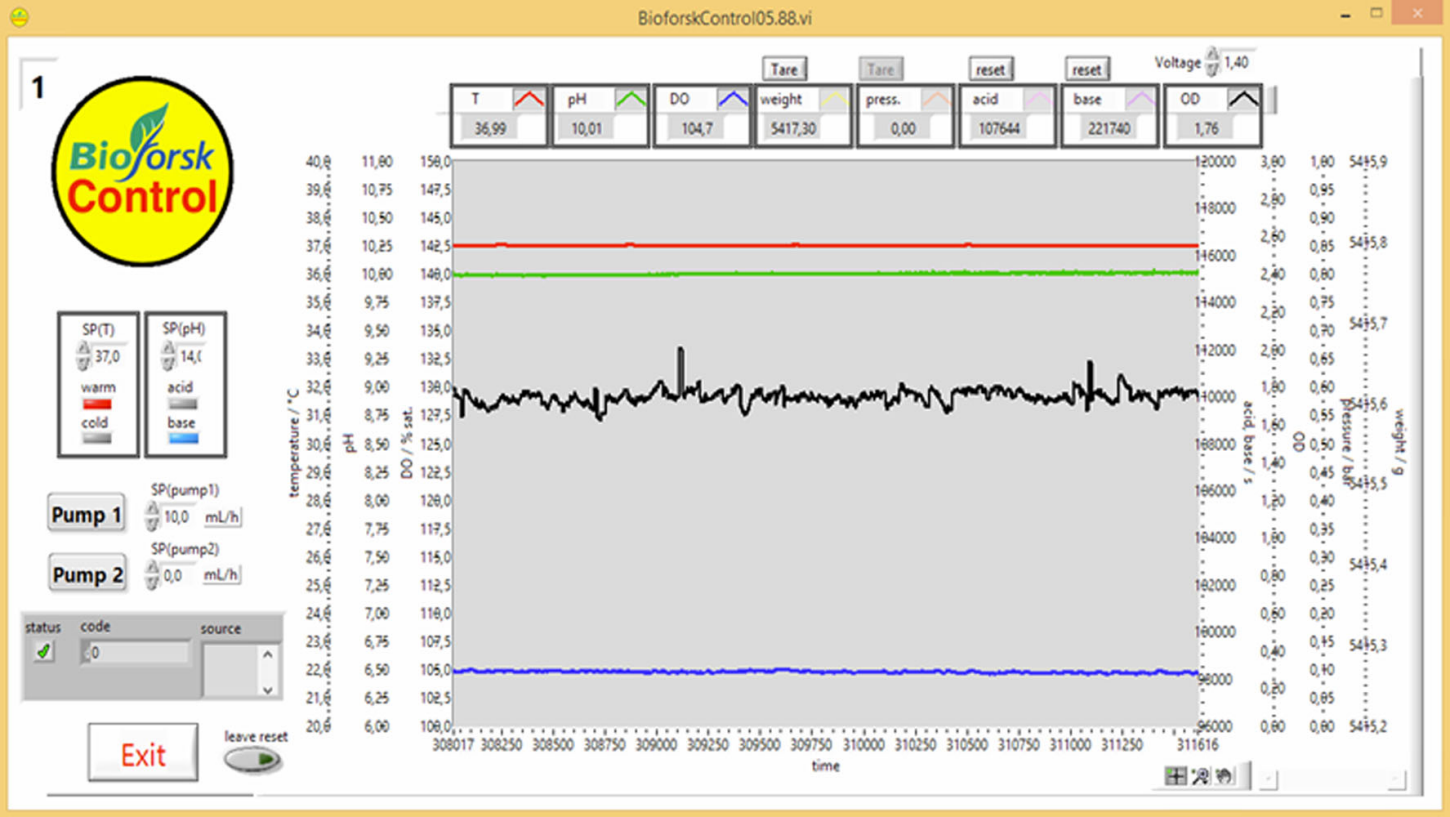
Screenshot of Bioforsk Control software

**Fig. 19:**
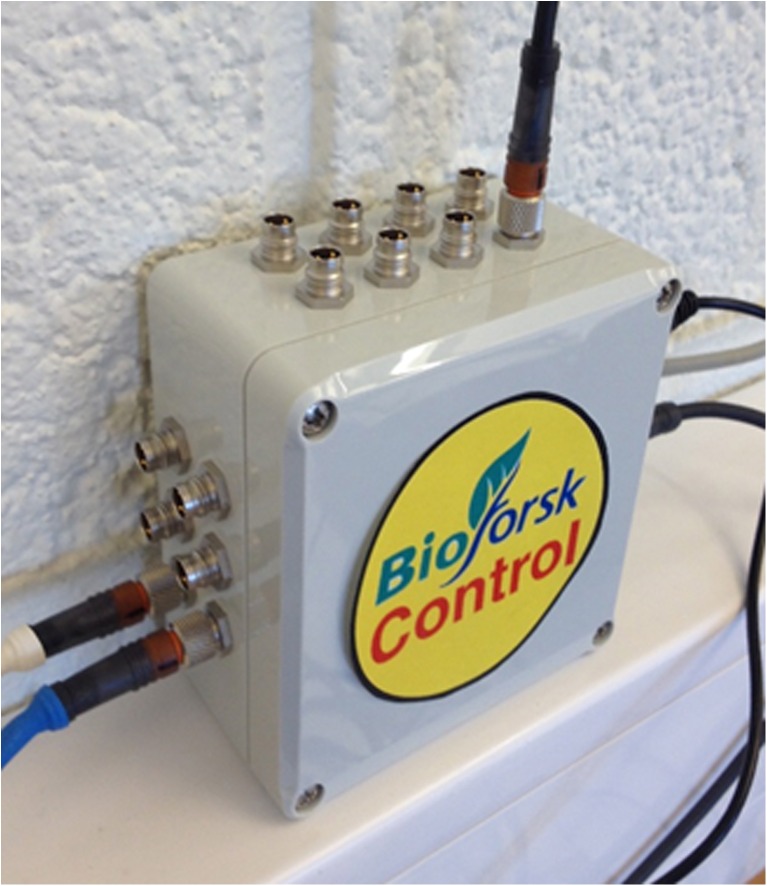
Control box of “Bioforsk Control”

With this setup, the DO and the weight (by hydrogen displaced water or feed bottle) could be measured, and the temperature, the pH, the OD, and the pump rate could be controlled. All data were numerically and graphically visualized on the computer screen (updated every 5 sec, history for last 5 h, see Fig. 18) and saved in a .txt file every minute. The USB-6008 AD-converter, the USB-RS485-Modbus converter, and the power supply for the sensors were integrated into a small control box (Fig. 19) so that all sensors and actuators (other than the balance and pump) could conveniently be connected to the computer system.

### Summary

The status in the field of photobioreactors used for hydrogen production from microalgae has been summarized, with an emphasis on agitation principles. A photobioreactor designed for the purpose of microalgae cultivation and hydrogen production has been thoroughly described. The photobioreactor consists of a culture vessel with a flat plate design, a stand with a motor for agitation of the culture, a gas collection system, and a control system. The flat plate format is widely considered to have many advantages for use in algae cultivation compared to the other designs described above, such as even light path and efficient mixing and gas exchange through sparging. We have shown that these advantages can be combined with an adequate design for hydrogen production, where the flat plate culture vessel is placed in a horizontal position and moved with a rocking motion for agitation and gas exchange. The rocking motion of a horizontal flat panel leads to a large surface area between the culture and headspace, even though the headspace volume inside the reactor can be quite small. This feature is an advantage for the release and collection of the hydrogen gas, compared to the other designs described initially. The importance of material choices for photobioreactors to be used for hydrogen production from microalgae has also been described in detail, both in respect to strength, convenience, hydrogen tightness, and possibly negative effects of materials on algae cells. The importance of the latter has, to our experience, been understated in the literature. The most significant challenge in the development of the described system has been to combine a culture vessel in motion with hydrogen collection, without leakage of the hydrogen gas. The solution was to mount the collection unit directly on the culture vessel, as described above, in order to avoid that any connections in movement were exposed to the hydrogen gas. The custom-built control system was designed to log and control temperature, pH, and optical density and additionally log the amount of produced gas and dissolved oxygen concentration. In summary, specific challenges related to photobioreactors designed for hydrogen production from microalgae have been addressed, and an operational photobioreactor for this purpose has been constructed.
